# Therapeutic Dilemma of a Juxtapapillary Retinal Capillary Hemangioma

**DOI:** 10.7759/cureus.76598

**Published:** 2024-12-29

**Authors:** Nurul Munirah Mohamad, Siti Sarah Shokri, Fatimah Suhaila Sukaimy, Wan Norliza Wan Muda, Liza Sharmini Ahmad Tajudin

**Affiliations:** 1 Department of Ophthalmology and Visual Science, Universiti Sains Malaysia School of Medical Sciences, Kubang Kerian, MYS; 2 Department of Ophthalmology and Visual Science, Hospital Tengku Ampuan Afzan, Kuantan, MYS; 3 Department of Ophthalmology and Visual Science, Ophthalmology Clinic, Hospital Universiti Sains Malaysia, Universiti Sains Malaysia, Kubang Kerian, MYS

**Keywords:** anti-vegf therapy, capillary hemangioma, exudative retina detachment, juxtapapillaryretina, pars plana vitrectomy, photodynamic therapy (pdt), von hippel-lindau disease (vhl)

## Abstract

A juxtapapillary retinal capillary hemangioma (JRCH) is a rare vascular hamartoma located on the optic nerve head or adjacent region. While often associated with von Hippel-Lindau (VHL) disease, JRCHs can also occur as an isolated condition, presenting unique therapeutic challenges and risks of visual impairment. We report a case of a 50-year-old Malay gentleman with diabetes mellitus who presented with a non-progressive superior visual field defect in his left eye for three months. Fundus examination revealed a raised reddish mass adjacent to the nasal optic disc, accompanied by macular exudates. Optical coherence tomography confirmed cystoid macular oedema, while fundus fluorescence angiography revealed feeder and draining vessels with late leakage and adjacent small vessel vasculitis. Systemic evaluation found no evidence of VHL or other abnormalities. Despite treatment with photodynamic therapy and intravitreal anti-vascular endothelial growth factor (anti-VEGF) injections, the patient’s vision progressively deteriorated. This case highlights the diagnostic and therapeutic challenges associated with JRCH and emphasizes the need for early recognition and tailored interventions to mitigate significant visual impairment.

## Introduction

A juxtapapillary retinal capillary hemangioma (JRCH) is a rare vascular retinal hamartoma that is typically located at or immediately adjacent to the optic nerve head. This lesion can present as an isolated finding or in association with von Hippel-Lindau (VHL) disease, an autosomal dominant tumor syndrome [1,2]. VHL disease is characterized by the development of both benign and malignant tumors in multiple organs, including the eyes, central nervous system, kidneys, pancreas, and adrenal glands. JRCHs associated with VHL disease are often diagnosed at a younger age compared to those that occur sporadically. Isolated JRCHs, however, are rarely reported in the literature and are not associated with the systemic manifestations of VHL [3]. First described in 1912, JRCHs are considered a diagnostic and therapeutic challenge for ophthalmologists due to their proximity to the optic nerve. The vascular nature of these lesions increases the risk of complications such as macular edema, exudation, and tractional retinal detachment, all of which may significantly impair visual function. In contrast to peripheral retinal hemangiomas, which are more frequently encountered in VHL patients, JRCHs are less common and require individualized management strategies due to their location near the optic disc [4]. To date, only 58 cases of isolated JRCHs have been documented in the literature, with reports spanning from their initial description in 1912 through to 1975. These lesions are most often diagnosed in older adults, distinguishing them from the JRCHs associated with VHL, which are typically detected earlier in life. The rarity of isolated JRCHs has contributed to limited knowledge regarding their natural disease course and optimal management strategies. Current treatment approaches include observation, laser photocoagulation, anti-vascular endothelial growth factor (anti-VEGF) therapy, photodynamic therapy, and surgical interventions, each with varying degrees of success [4,5]. The therapeutic dilemma in managing JRCHs arises primarily from their location near the optic nerve, where intervention poses significant risks to central vision. Balancing the potential benefits of treatment against the risk of collateral damage is critical, especially in cases where the lesion is asymptomatic or minimally symptomatic. Furthermore, the decision to treat may depend on the presence of associated complications, such as exudation or macular edema, that threaten vision. Here, we report a case of isolated JRCH, describing the clinical presentation, disease course, and management strategies. This case contributes to the limited body of literature on isolated JRCHs and underscores the challenges in achieving optimal outcomes for this rare retinal pathology.

This article was previously presented as a meeting abstract at the 37th Singapore-Malaysia Joint Meeting in Ophthalmology, held in Singapore from January 19 to 21, 2024.

## Case presentation

A 50-year-old Malay gentleman with underlying diabetes mellitus and hypertension presented with a non-progressive superior visual field defect in the left eye (LE) for a duration of three months. His ocular and systemic history was unremarkable, with no family history of VHL disease.

**Figure 1:**
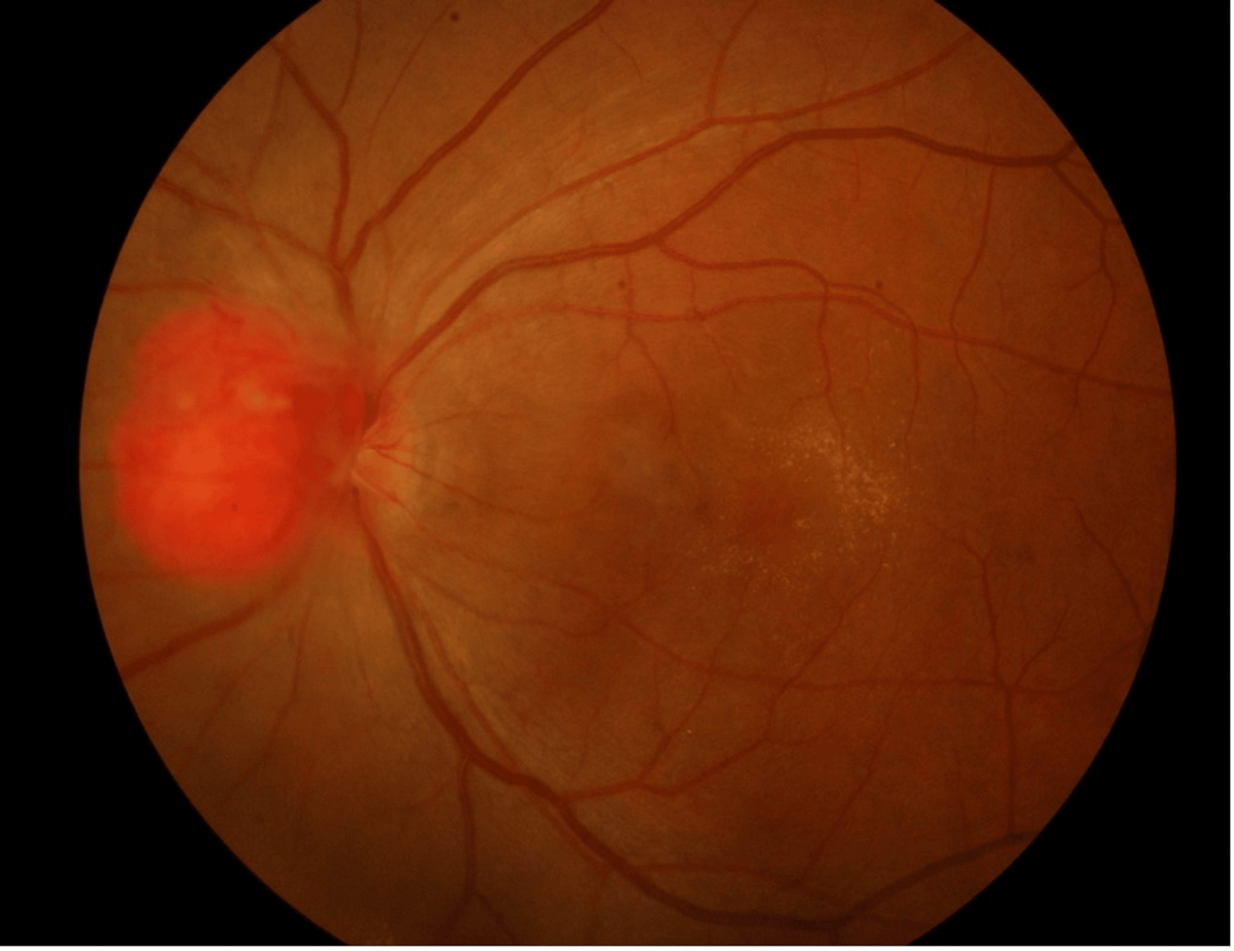
Fundus image of the left eye (LE) showing a focal, raised reddish mass at the nasal optic disc, measuring two-disc diameters (DD), with associated macular exudates.

At presentation, the best-corrected visual acuity (BCVA) was 6/9 in the right eye (RE) and 6/60 in the LE, accompanied by reduced optic nerve function in the LE and a positive relative afferent pupillary defect (RAPD). Anterior segment slit lamp examination of both eyes was unremarkable. Dilated fundus examination of the LE revealed a focal, raised reddish mass at the nasal optic disc, measuring two-disc diameters (DD), with associated exudates at the macula (see Figure 1). No retinal hemangiomatous lesions were detected in the periphery or the RE.

**Figure 2:**
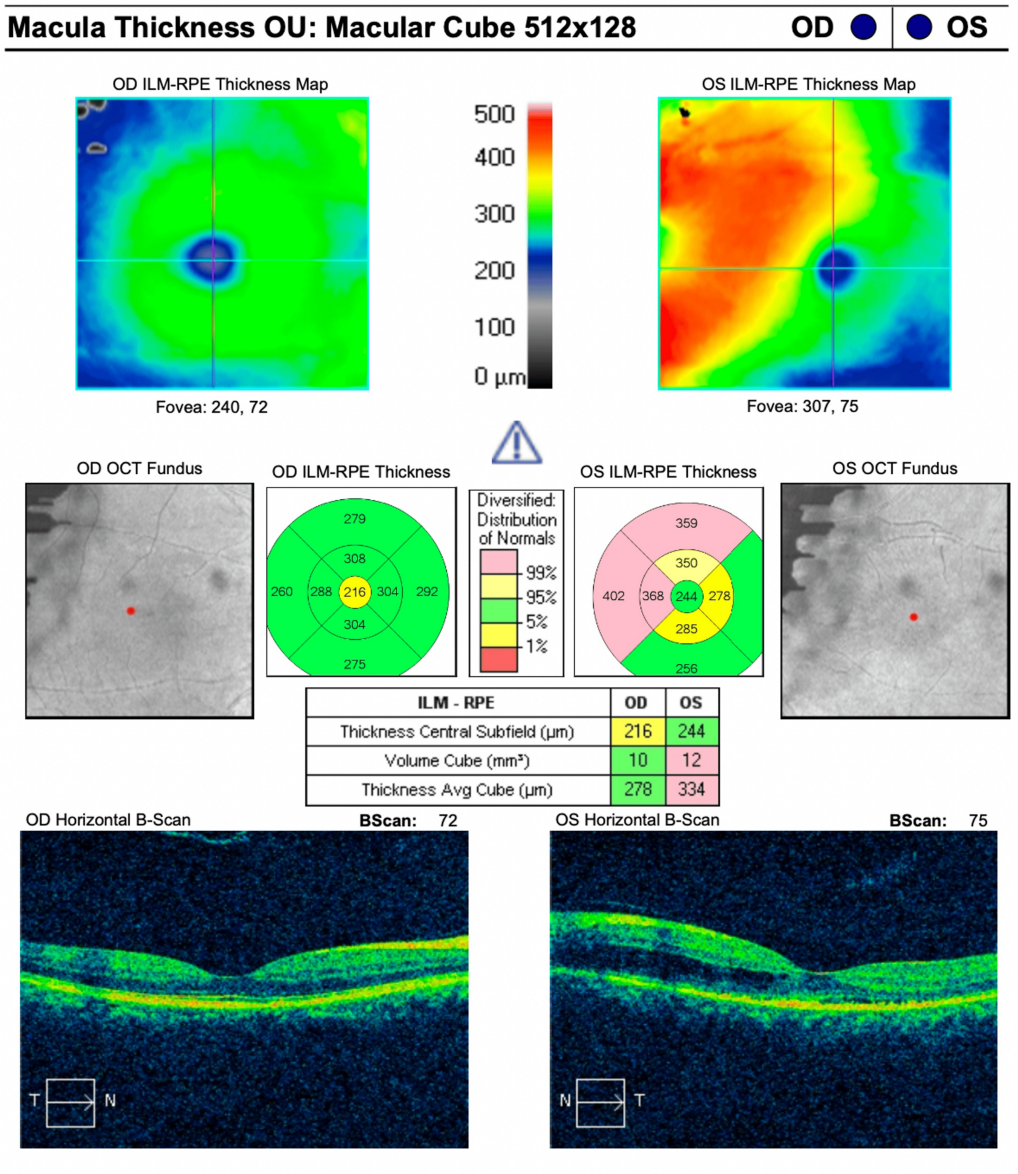
Optical coherence tomography (OCT) image of the RE shows no evidence of SRF or IRF, while the OCT image of the LE reveals the presence of both IRF and SRF. RE: right eye, SRF: subretinal fluid, IRF: intraretinal fluid, OCT: optical coherence tomography, LE: left eye

Optical coherence tomography (OCT) of the macula revealed the presence of intraretinal fluid (IRF) and subretinal fluid (SRF) in the LE (see Figure 2).

**Figure 3:**
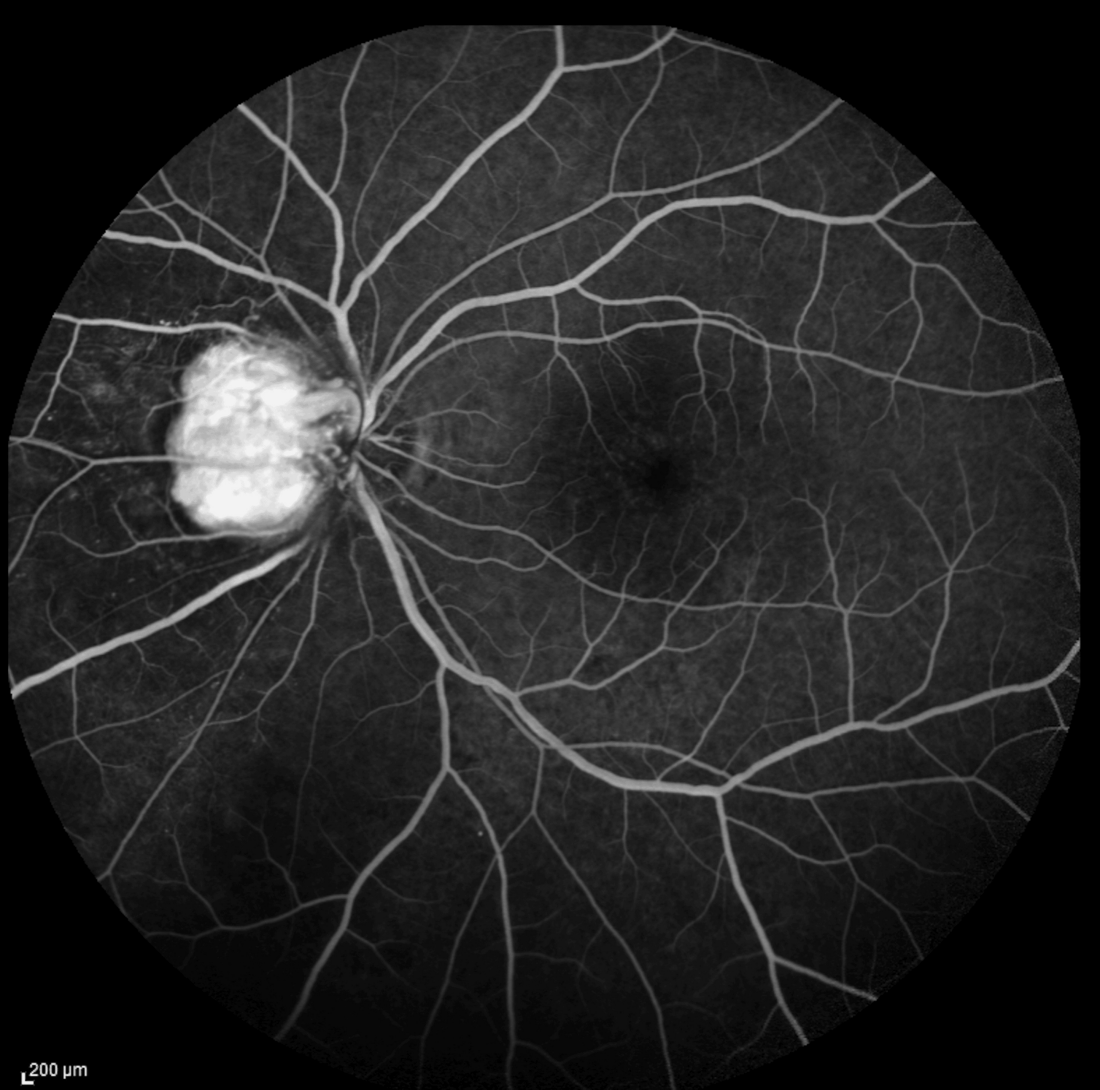
Fundus fluorescence angiography (FFA) showing a short feeder vessel and draining vessel, with adjacent small-vessel vasculitis and late leakage from the lesion.

Fundus fluorescence angiography (FFA) revealed short feeder vessels and draining vessels with adjacent small-vessel vasculitis, along with late leakage from the lesion. No peripheral capillary dropout, vasculitis, or choroiditis was observed (see Figure 3).

Other systemic evaluations, including a CT brain scan, Mantoux test, and urine and blood analyses, were conducted, all of which yielded unremarkable results. Based on the findings, a diagnosis of isolated exophytic juxtapapillary capillary hemangioma was established.

**Figure 4:**
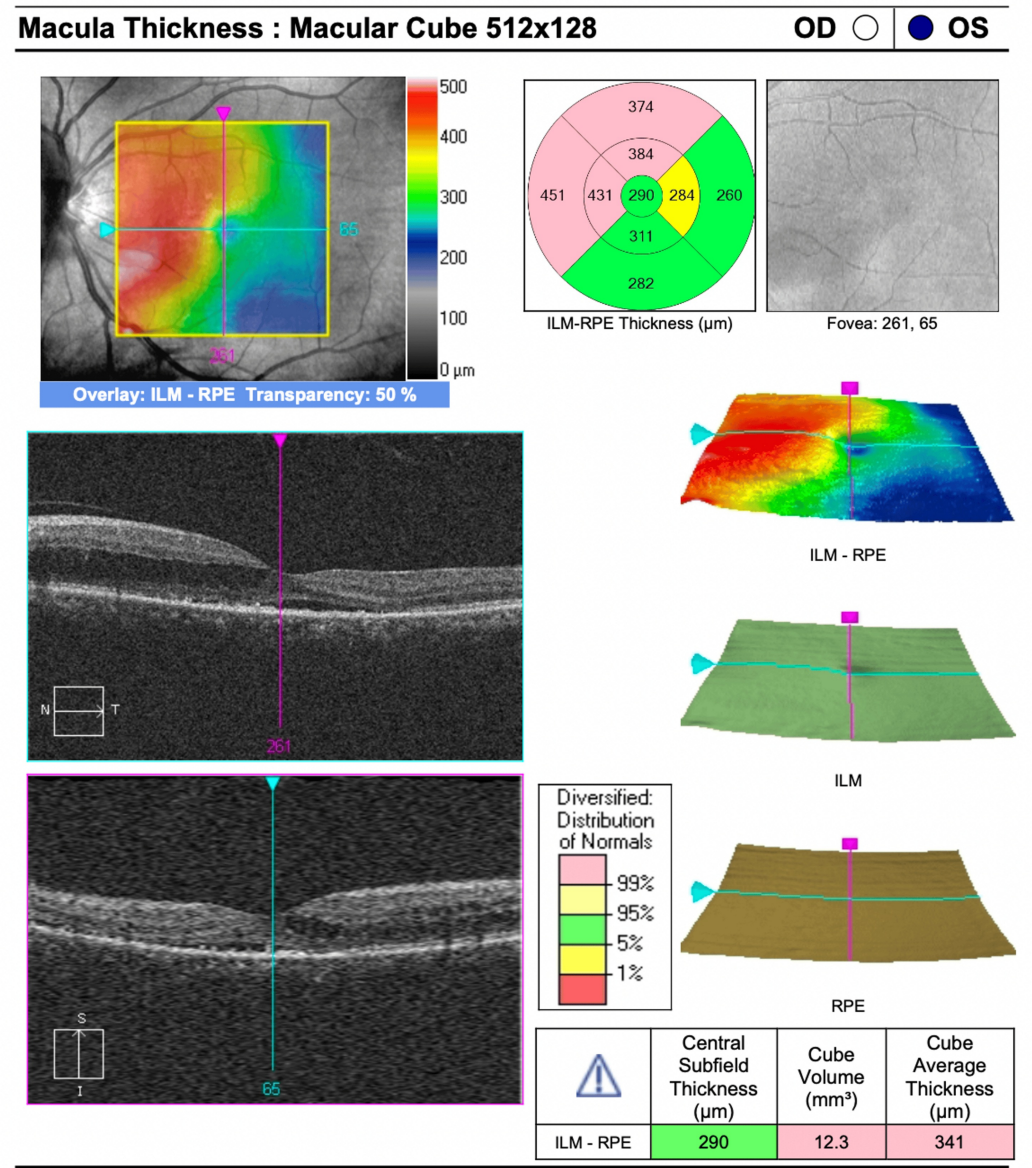
Optical coherence tomography (OCT) macula in the LE after initial PDT and intravitreal ranibizumab injection. LE: left eye, PDT: photodynamic therapy

**Figure 5:**
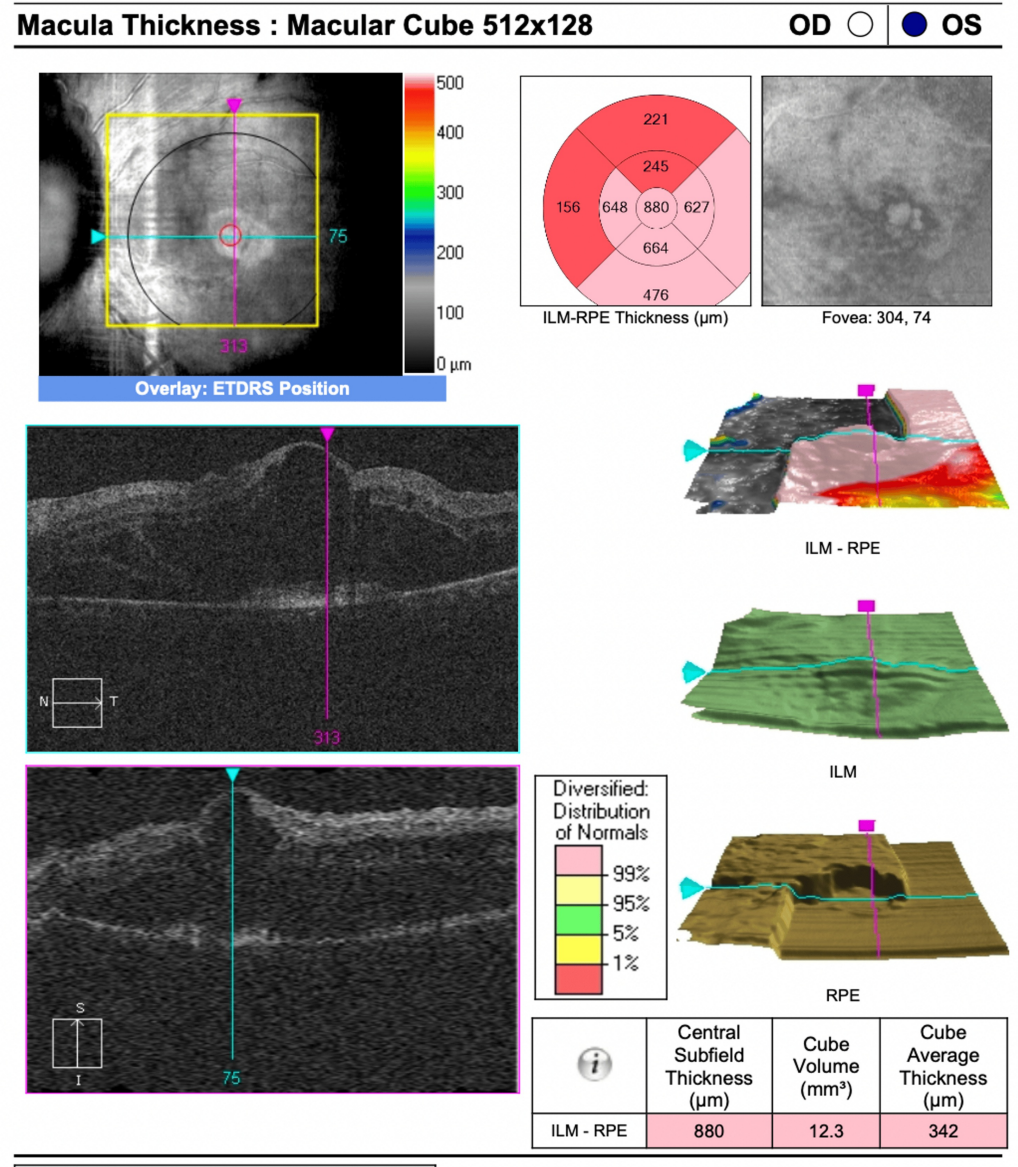
Optical coherence tomography (OCT) macula in the LE after multiple treatment modalities showed increased IRF and SRF. LE: left eye, IRF: intraretinal fluid, SRF: subretinal fluid

A combination of photodynamic therapy (PDT) and intravitreal anti-VEGF injection was initiated promptly. Following initial treatment, the best-corrected visual acuity (BCVA) in the LE improved to 6/15 but gradually declined over time. OCT of the macula demonstrated increasing IRF and SRF despite multiple treatment sessions (Figures 4, 5).

The lesion expanded in size, covering the optic nerve head and leading to surrounding exudative retinal detachment. The patient also underwent several sessions of laser photocoagulation (PRP). However, the patient eventually experienced loss of visual acuity in the LE with no light perception vision (NPL) despite multiple treatment modalities. Throughout the follow-up, the fellow eye fortunately remained free from the lesion.

## Discussion

JRCHs are therapeutic dilemmas for ophthalmologists due to their proximity to the optic disc. Given the specific location of these tumors, it is essential to rule out the potential of other optic nerve and retinal pathologies such as anterior ischemic optic neuropathy, papillitis, granulomatous disease, peri-papillary choroidal neovascularization, choroiditis, retinoblastoma, or retinal macroaneurysms [2].

Anterior ischemic optic neuropathy (AION) typically presents as sudden, painless vision loss with a pale or swollen optic disc but lacks the vascular features seen in JRCHs. It is often associated with systemic conditions such as hypertension and giant cell arteritis. Papillitis, or optic neuritis, presents with optic disc hyperemia, pain in eye movement, and systemic symptoms. Unlike JRCHs, papillitis is linked to demyelinating diseases such as multiple sclerosis and does not exhibit vascular tumor characteristics.

Retinal macroaneurysms, typically found along retinal arteries and associated with hypertension, lack the optic disc location and vascular tumor features of JRCH. Granulomatous diseases such as sarcoidosis or tuberculosis may cause optic disc swelling and peripapillary changes. Systemic investigations, including serum ACE levels and imaging, can help differentiate these conditions. Choroiditis, an inflammatory condition of the choroid, may involve the optic nerve and cause peripapillary swelling, but it lacks the vascular appearance of JRCH.

In children, retinoblastoma must be considered. This malignant retinal tumor often presents as a white elevated mass near the optic nerve, with calcifications seen on imaging that are absent in JRCHs. Lastly, peripapillary retinal detachment can mimic JRCHs with subretinal fluid but is distinguishable on OCT, which shows the characteristic retinal separation. Diagnosis of JRCH is facilitated by fluorescein angiography, which highlights feeder and draining vessels, and OCT, which identifies structural abnormalities. Systemic evaluations may be necessary to rule out associated systemic diseases. Combining clinical examination, imaging, and systemic workup ensures accurate differentiation of JRCHs from other conditions.

Therefore, a thorough history, examination, and ancillary testing play a crucial role in differentiation. It has been proposed that a significant number of these tumors, possibly 43% to 50%, may also present with peripheral retinal lesions, commonly located at the temporal, inferotemporal, and superotemporal regions [3-6]. As a result, a comprehensive examination of the peripheral retina is recommended. During the arterial phase of fluorescein angiography, the vasculature over the tumors becomes visible, showing diffuse hyperfluorescence, and in the late phase, staining of the hemangiomas occurs as the dye extravasates into the surrounding edematous tissue. Indocyanine green angiography also reveals dye leakage associated with vascular tumors. Juxtapapillary hemangiomas exhibit three distinct growth patterns: endophytic, exophytic, and sessile forms [2,3]. The most clinically noticeable are the endophytic types-smooth, elevated lesions that extend over the surface of the nerve and adjacent retina, protruding into the vitreous cavity. The endophytic type, especially when bilateral, has been linked to VHL disease and is associated with poorer visual acuity [4,5]. Exophytic tumors present as nodular, orange-colored lesions growing in the outer layers of the retina. This variant is less well-defined and can be challenging to distinguish from metastatic tumors, malignant melanoma, or choroidal hemangiomas. Lastly, sessile tumors are flat lesions with subtle and indistinct margins that develop in the intraretinal layers. The clinical course of JRCHs varies, generally showing a gradual decline in vision. These lesions may remain stable or grow slowly, preserving good visual acuity for an extended period. Although spontaneous regression has been documented, such events are rare [7-9]. The ongoing growth of the tumors, whether treated or not, poses a risk of exudative decompensation. As the tumors enlarge, the capillaries within the hemangiomas may lose their integrity, leading to progressive intraretinal and subretinal macular exudation [7-9]. Other ocular complications associated with JRCHs include the formation of epiretinal membranes (ERM), serous or tractional retinal detachment (TRD), subretinal hemorrhage, or the development of peripheral tumors in either eye [10]. Chronic complications may lead to iris neovascularization or glaucoma, and in cases of severe pain, enucleation may be required.

Although there are no definitive guidelines for management, recommended treatment criteria have been suggested by Gracia-Arumi J et al. [10] in 2000, which involve close monitoring during the early stages of the disease, with treatment initiation as the lesion progresses or vision is compromised. The goal is not to obliterate the tumor but to control any sight-threatening complications such as exudation, subretinal fluid accumulation, macular edema, exudative retinal detachment, glial proliferation with ERM formation, TRD, and visual deterioration [11-13]. Very rarely, these tumors may resolve spontaneously. Treatment options for JRCHs include laser photocoagulation, brachytherapy, transpupillary thermotherapy, photodynamic therapy (PDT), and surgical excision, though none have been proven effective in reducing tumor progression [14,15].

PDT and intravitreal anti-VEGF therapy have recently been reported as safe for the optic nerve and enable more selective vascular occlusion. According to Ziemssen F et al. [12] in 2007, anti-VEGF or PDT alone as monotherapy is less effective than combined therapy. The rationale behind anti-VEGF therapy is to regress tumor growth and reduce exudation, while PDT, using verteporfin, offers more selective vascular occlusion, causing fibrosis and the involution of small tumors [14,15]. However, there are limitations with PDT: it is less effective in larger tumors (as reactive oxygen species cannot penetrate deeper vessels) and can cause fibrosis with the progression of ERM, transient optic disc edema, retinal vessel occlusion, optic neuropathy, vitreous hemorrhage, massive retinal detachment, and hemorrhage [14-16].

Vitreoretinal surgery may be considered a treatment option for the endophytic type, especially if associated with ERM formation, serous or tractional retinal detachment, or vitreous hemorrhage [17]. Some studies have reported successful surgical outcomes when combined with PDT and/or anti-VEGF therapy before and after surgery. Gaudric A et al. [17] in 2011 suggested that surgery may be preceded or followed by PDT, while Mariotti C et al. [18] in 2014 reported a successful case of progressive paramacular JRCHs, with sessile exophytic growth and TRD treated with two sessions of PDT after surgery. Fong AH et al. [19] in 2011 reported a successful case of inferotemporal JRCHs with TRD at the macula treated with combined intravitreal anti-VEGF and PDT one week prior to surgery. Larger-scale studies are necessary to establish a successful treatment strategy for JRCHs.

## Conclusions

JRCHs are a rare variant of retinal capillary hemangiomas. A thorough history, examination, and ancillary investigations are essential for the diagnosis and treatment of JRCHs. It is also important to note that each case of JRCH is unique, and the optimal treatment plan depends on the patient and the specifics of their condition. Currently, there is no consensus regarding the treatment options for JRCH. The management of these patients remains challenging, and a combination of treatments or a tailored approach may be necessary to achieve the best outcome for the patient.
